# Can Acute Exercise Lower Cardiovascular Stress Reactivity? Findings from a Scoping Review

**DOI:** 10.3390/jcdd9040106

**Published:** 2022-03-31

**Authors:** Wei Joo Chen, Arimi Fitri Mat Ludin, Nor M. F. Farah

**Affiliations:** 1 Center for Community Health Studies, Faculty of Health Sciences, Universiti Kebangsan Malaysia, Kuala Lumpur 50300, Malaysia; p110742@siswa.ukm.edu.my; 2 Center for Healthy Ageing and Wellness, Faculty of Health Sciences, Universiti Kebangsan Malaysia, Kuala Lumpur 50300, Malaysia; arimifitri@ukm.edu.my

**Keywords:** aerobic exercise, HIIT, autonomic function, blood pressure, heart rate, chronic stress

## Abstract

Exaggerated cardiovascular reactivity to and delayed recovery from stress increase the risk of cardiovascular diseases in the future. While exercise training has been shown to attenuate stress-induced cardiovascular reactivity and enhance recovery from stress, the effects with acute exercise are less characterized. The aim of this scoping review was to explore the range and characteristics of published evidence regarding acute exercise on cardiovascular reactivity and stress recovery. The secondary objective was to highlight research gaps and implications for future research. A total of 36 articles met the review inclusion/exclusion criteria, involving 1200 participants from various age groups, fitness and health status. Blood pressure (BP) reactivity was the most measured outcome, followed by heart rate (HR) reactivity, and to some extent, heart rate variability. Overall, acute exercise particularly of the moderate-intensity aerobic type effectively reduced stress-induced BP reactivity in the general population. The effects on HR reactivity and cardiovascular recovery were inconclusive. Further research would be recommended to establish if other forms of exercise intensity or type are equally beneficial to lower exaggerated cardiovascular responses to stress. Despite methodological differences and limitations, the available evidence supports the therapeutic potential of acute exercise in addressing the ill effects of stress on cardiovascular health.

## 1. Introduction

Acute exposure to a psychological or physical stressor evokes heightened cardiovascular responses, characterized by rises in heart rate (HR) and blood pressure (BP) from a baseline or resting state, termed as cardiovascular reactivity. Over the short term, such stressor-induced cardiovascular reactivity provides hemodynamic and metabolic support to enable animals and humans to adapt to life-threatening or challenging situations (i.e., fight-or-flight response). The key pathways responsible for stress-induced cardiovascular reactivity are the hypothalamic–pituitary–adrenal (HPA) axis and the autonomic nervous system outflow to the heart and vasculature, resulting in changes to cardiovascular parameters such as cardiac output, HR, vascular resistance and ultimately BP [1]. Over the long term, however, frequent exposures to physical and/or psychological situations in day-to-day life can result in tendencies to exhibit exaggerated cardiovascular reactivity and delayed recovery, which can adversely impact homeostasis and trigger or exacerbate an array of pathophysiological changes involving the cardiovascular system [2,3]. This is so much so that individuals showing exaggerated or large magnitudes of cardiovascular reactions in response to a stressor are at greater risk for premature development of hypertension and other precursors to coronary heart disease, adverse clinical cardiovascular events and premature cardiovascular mortality [3,4]. As such, understanding how cardiovascular reactivity can be prevented or mitigated may be relevant in stress management and lowering the risk of cardiovascular disease (CVD).

The benefits of exercise on cardiovascular health are well-established. Many studies have shown that regular physical activity and exercise training are generally associated with better cardiovascular responses to acute stress, and not only does exercise attenuate the magnitude of hyperarousal associated with the stress response but also enhances cardiovascular recovery after stress exposure [2,5]. Exercise also exerts beneficial effects on the HPA axis functioning by lowering stress-induced cortisol responses and hastening HPA recovery via a negative feedback loop [6]. For example, Klaperski et al. [7] showed that subjects who underwent 12 weeks of endurance exercise training had significantly reduced stress reactivity to a psychosocial stressor as measured by cortisol, HR and heart rate variability (HRV) responses when compared to a wait-list control group. Other reports indicated that exercise training is associated with enhanced parasympathetic tone, reduced sympathetic nerve activity and improved HRV [8], which may lead to better cardiac autonomic control in response to stress. Apart from exercise training, there are also reports showing that even a single or an acute bout of exercise can transiently mitigate the exaggerated cardiovascular responses to stressful conditions [9,10,11,12,13]. The evidence indicates that the beneficial effects of exercise in lowering stress-induced cardiovascular reactivity can be achieved acutely and without having to undergo extensive exercise training.

However, the evidence surrounding acute exercise and cardiovascular reactivity is still not well characterized, due to the huge variability in exercise protocols, e.g., intensity, type and duration. Further, the usage of different types of stressor protocols to induce stress in the laboratory adds a challenge in the interpretability of research findings. These huge variations in methodology have led to inconsistencies in the study findings, which limits our current understanding surrounding the role of acute exercise in minimizing cardiovascular hyperarousal related to stress response. Furthermore, with the increasing global prevalence of chronic stress especially since the coronavirus pandemic outbreak, recognizing exercise protocols that are effective to offset cardiac hyperarousal states associated with daily stress is useful to help mitigate abnormal cardiovascular responses or events in the long term, especially among those who are vulnerable to stress exposures. 

As such, this scoping review intended to explore the range and variety of available studies using acute exercise as a therapeutic tool to lower stress-induced cardiovascular reactivity in the general population and to produce evidence of additional benefits of acute exercise on cardiovascular health beyond improving the traditional risk factors. The primary objective of this scoping review is to catalogue research evidence from primary peer-reviewed published studies investigating the effects of acute exercise with different modalities and intensities on stress-induced cardiovascular reactivity. A secondary objective is to identify key gaps in the literature for future research.

## 2. Methods

We used the 5-stage methodological framework by Arksey and O’Malley [14] and Levac et al. [15] and guided by the Preferred Reporting Items for Systematic Reviews and Meta-Analyses (PRISMA) scoping review extension checklist [16] (Appendix A).

### 2.1. Identifying the Research Question

Research question formulation was guided by item 4 in the PRISMA scoping review extension checklist. The research questions developed for this review were: “What is known from published evidence about the effects of acute exercise on cardiovascular reactivity and recovery?” and “What are the current knowledge gaps?”. Cardiovascular reactivity is defined as the degree of change in cardiovascular variables (e.g., BP, HR, HRV) in response to an acute exposure of psychological or physiological challenge or stressor. Cardiovascular recovery can be defined as: (i) how long the variables remained elevated after removal of stressor, or (ii) the degree of change in variables after the stressor is removed in relation to during stress exposure. The cardiovascular variables focused on in this review include BP, HR and HRV.

### 2.2. Identifying Relevant Studies

The literature was searched in four electronic databases: PubMed, EBSCO Medline, Scopus and Web of Science. The search strategy used keywords related to “acute exercise” paired with at least one cardiovascular outcome term. Specifically, keywords such as “aerobic exercise”, “resistance exercise”, “high intensity interval”, “moderate intensity” and qualifiers for cardiovascular outcomes such as “blood pressure reactivity”, “heart rate variability”, “sympathetic” and “autonomic” were used. Appropriate truncation symbols were used to account for search term variations. Searches were conducted for papers published up to December 2021. All citations were exported to EndNote, where duplicates were identified and removed. Thereafter, the remaining articles were exported to an online management system Rayyan [17]. Articles were subsequently assessed by title and abstract by two authors. As an additional search strategy, the reference lists of relevant review papers were scanned for additional articles, but the review papers were not included.

### 2.3. Study Selection

Titles and abstracts were screened according to the inclusion and exclusion criteria, with some cross-checking performed early in the process to assess agreement between authors. The inclusion criteria were: (1) any studies using acute exercise protocol of any modality or type, performed in single session and lasting any duration, (2) studies employing acute laboratory stressor protocol (physiological or psychological in nature) that lasted 1 h or less to induce cardiovascular reactivity and/or recovery, and must be performed after exercise, (3) studies that included at least one measurement for stress-induced cardiovascular reactivity and/or recovery, (4) studies involving human subjects of any age group, sex, BMI and physical activity levels. Articles were excluded if: (1) exercise protocols were of training type or long term, (2) exercise protocol was combined with other intervention(s) where differentiating the individual effect of exercise was not possible (e.g., combined with deep breathing exercise), (3) exercise protocol involved stressors that were not laboratory-based (e.g., real class examinations, caregiving), (4) studies that have not undergone full peer review, (5) studies published in languages other than English. Full texts were subsequently retrieved and reviewed independently by two authors (WJC, NMFF). If one or both of the reviewers deemed an article to be potentially ineligible, the full texts of the relevant articles were examined and resolved by discussion. Reasons for excluding articles during the initial abstract and full text screening procedures were recorded.

### 2.4. Charting the Data

One author (WJC) extracted and charted the following data from each article: author(s), year of publication, subject characteristics (sex, age, sedentary/physically active, health status, smoking status), characteristic of exercise protocol (type, intensity, duration), method of inducing acute stress (physiological, cognitive or social), time of outcome measurement and outcome parameters (BP, HR, HRV reactivity and/or recovery). Exercise protocol or modalities were classified as either high-intensity interval exercise (HIIE), aerobic continuous exercise (ACE), resistance exercise (RE) or flexibility exercise (FE). Using the American College of Sports Medicine’s exercise intensity recommendations [18], protocols performed at 45–63% of VO_2_max (64–75% of HRmax) were classified as moderate intensity, protocols performed at 64–90% of VO_2_max (76–95% of HRmax) were classified as vigorous, while protocols performed at ≥91% of VO_2_max (≥96% of HRmax) were considered near maximal to maximal. The corresponding author (NMFF) checked the extractions. 

### 2.5. Collating, Summarizing and Reporting the Results

The results were presented in two ways: (a) a descriptive analysis to highlight the prevailing themes emerging from the charting process, and (b) a narrative summary of the key findings. 

## 3. Results

### 3.1. Selection of Included Studies

**Figure 1:**
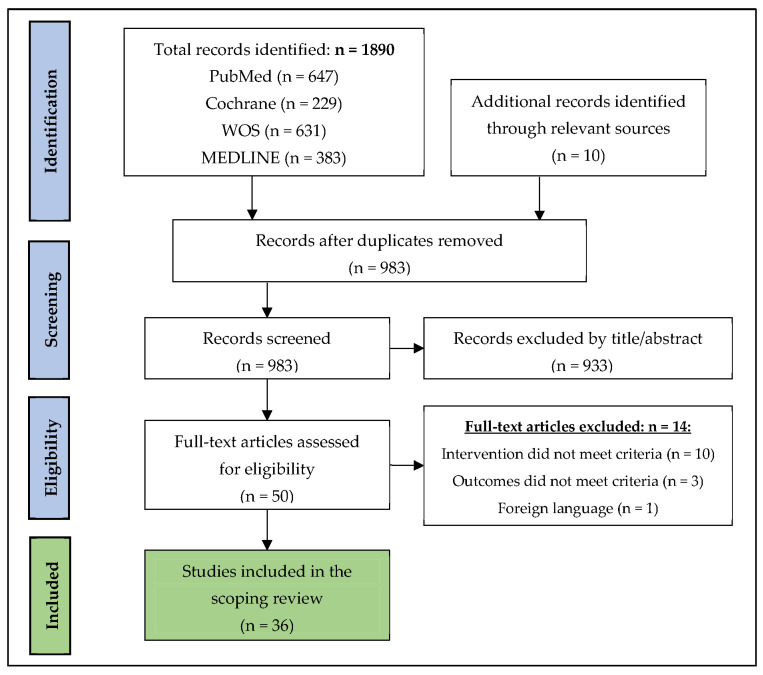
PRISMA flow diagram showing identified, included and excluded studies.

The main goal of our selection strategy was to identify articles that described the effects of an acute session of exercise on cardiovascular reactivity, namely BP, HR and HRV in the general population. The selection process is presented in a PRISMA flow diagram in Figure 1. The search results from the databases yielded 1890 papers. Ten papers were added from the reference list of relevant review papers [19,20]. After removing the duplicates, 983 papers were examined based on the title and abstract, during which 933 papers were excluded. A total of 50 papers were retained for full-text assessments, of which 14 papers were subsequently removed. Ultimately, a total of 36 papers fulfilled the inclusion criteria for this review and are presented in Table 1.

### 3.2. General Characteristics of Included Studies

Study characteristics of the included studies are presented in Table 1. The majority of studies were conducted in the USA (n = 14), followed by Brazil (n = 7) and the United Kingdom (n = 5). Other countries included Canada, Australia, Cuba, Finland, Germany, Japan, Malaysia and Switzerland. The year of publications were mostly between 1989 and 2021. A total number of 1177 of participants (599 males and 558 females, 20 were not indicated) were recruited across all 36 studies (ranging from 8 to 90 participants per study), consisting of mostly healthy, active and inactive individuals. There were studies involving participants with hypertension (n = 3), knee osteoarthritis (n = 1) and 894G > T endothelial nitric oxide synthase genetic polymorphism (n = 1). Gender-wise, 19 studies involved both genders, 11 studies involved only males and 5 studies were female-only. The participants’ ages ranged between 7 to 77 years old. The study designs consisted of crossover, randomized control trial (RCT) (n = 21), parallel RCT (n = 11) and pre–post trial (n = 5). Notably, Roemmich et al. [41] conducted two trials (crossover and parallel RCT) in a single paper; the results were discussed separately. Participants in crossover RCTs served as their own control and participants in parallel RCTs were divided into control groups and intervention groups for comparison, whereas no control groups were employed in pre–post studies.

### 3.3. Acute Exercise Protocols

Of the 36 reviewed studies, most of them employed the aerobic continuous exercise (ACE) (n = 27, 75%) modality, encompassing a broad range of activities, including ergometer cycling, treadmill running and walking. This is followed by high-intensity interval exercise (HIIE) (n = 7) and resistance exercise (RE) (n = 4), and a smaller proportion of studies examined flexibility exercises (FE) (n = 2) and combination of RE and ACE (n = 1). Regarding exercise intensities, 21 studies used moderate intensity, while others ranged between light intensity (n = 4), vigorous (n = 13) and near-maximal to maximal (n = 6). Among the studies, eight studies employed varying exercise intensities and compared the effects of intensity on cardiovascular outcomes [11,13,30,32,37,44,46,48]. The exercise duration for ACE ranged from 10 min to 1 h, with a study carrying out up to 2 h of cycling [24]. The duration for HIIE ranged from 20 min to 1 h, and between 20 to 30 min for FE.

### 3.4. Acute Laboratory Stressor Protocols

The methods of inducing acute stress in these studies involved laboratory stressors typically used in stress reactivity research, and we categorized them into cognitive (i.e., mental arithmetic task, Stroop task or color–word interference task, recite digits backwards), physiological (i.e., cold pressor task, lower body negative pressure) and social (public speaking, interview) stressors. A majority of studies employed cognitive stressors (n = 26), while others used physiological stressors (n = 11) and social stressors (n = 10). Some of these studies used a combination of two or three stressor categories (n = 10). In most studies, participants were exposed to a laboratory stressor post-exercise, while in 10 studies, the exposure was conducted in pre- and post-exercise conditions. The time of exposure to a laboratory stressor ranged from immediately post-exercise to 24 h post-exercise.

### 3.5. Cardiovascular Outcomes

All studies reported stress-induced reactivity and/or recovery using systolic blood pressure (SBP), diastolic blood pressure (DBP), mean arterial pressure (MAP), HR and/or HRV. Measurements of these parameters were generally carried out during exposure to a laboratory stressor to assess cardiovascular reactivity, and up to 15 min post-exposure for cardiovascular recovery.

#### 3.5.1. SBP Reactivity

SBP reactivity was assessed in 33 studies, with 17 (52%) studies showing significant reductions in SBP reactivity following exposure to an acute stressor with exercise when compared with either control (n = 14) or pre-exercise (n = 3) conditions. A majority of these studies employed the ACE protocol (n = 14), followed by HIIE (n = 3), RE (n = 2) and RE + ACE (n = 1). The exercise intensities ranged between light (n = 2), moderate (n = 9), vigorous (n = 7) and near-maximal to maximal (n = 2). Two of these studies were conducted among hypertensive subjects [9,25]. Five studies compared the effects of exercise on SBP reactivity between moderate and vigorous intensities. Rejeski et al. [37] and Steptoe et al. [48] showed only exercise in vigorous intensity (both protocols were ACE) produced significant reductions. Alderman et al. [11] showed that though both exercise intensities (both ACE protocol) lowered SBP reactivity, the effect was more pronounced with vigorous intensity compared to moderate. Farah et al. [13] and Meireles et al. [30] compared between HIIE and moderate-intensity ACE protocols. The former showed both intensities attenuated SBP reactivity compared to control with no differences between the two, while the latter showed both intensities do not produce significant reductions.

#### 3.5.2. DBP Reactivity

DBP reactivity was assessed in 33 studies, with 20 (61%) studies showing significant reductions in DBP reactivity following exposure to an acute stressor with exercise when compared with either control (n = 16) or pre-exercise (n = 4) conditions. The reductions were observed in a majority of studies employing ACE protocol (n = 15), followed by HIIE (n = 4), RE (n = 2) and RE + ACE (n = 1). The exercise intensities ranged between light (n = 2), moderate (n = 10), vigorous (n = 8) and near-maximal to maximal (n = 2). Three studies involved hypertensive subjects [9,25,51], and all reported attenuated DBP reactivity. In the three studies that compared the effects of moderate and vigorous intensities [11,13,37], no differences were observed between exercise intensities on DBP reactivity. Farah et al. [13] observed a reduction in DBP reactivity with the HIIE protocol but not with ACE. Based on the limited findings, whole-body resistance-based exercise (RE) [25,27,33] appeared to be more effective in lowering DBP reactivity than isolated RE [35]. 

#### 3.5.3. MAP Reactivity

Eleven out of sixteen studies showed significant reductions in stress-induced MAP reactivity following exercise when compared with either control (n = 8) or pre-exercise (n = 3) conditions. The reductions were reported in studies employing the ACE protocol (n = 10), followed by RE (n = 2) and HIIE (n = 1). The exercise intensities ranged between light (n = 2), moderate (n = 5), vigorous (n = 4) and near-maximal to maximal (n = 2). With regards to comparison between exercise intensities, only one study showed a greater attenuating effect of vigorous-intensity exercise compared to exercise with moderate intensity [37].

#### 3.5.4. HR Reactivity

HR reactivity was examined in 28 studies, yet only 7 (25%) studies showed significant reductions following exercise compared to either control (n = 6) or pre-exercise (n = 1). The reductions were observed in studies employing ACE (n = 7) and HIIE (n = 1) protocols with moderate (n = 4), vigorous (n = 3) and near-maximal to maximal (n = 1) intensities. One report showed lower HR reactivity following vigorous-intensity exercise when compared to moderate intensity, but this effect was only prominent five minutes post-exercise and soon disappeared as time progressed [11]. 

#### 3.5.5. Cardiovascular Recovery

Overall, 13 studies reported cardiovascular recovery outcomes (SBP, DBP, MAP, HR). Measurements for cardiovascular outcomes in the recovery period were conducted at various intervals, i.e., 2 min (n = 4), 3 min (n = 1), 5 min (n = 3), 10 min (n = 4) and 15 min (n = 1) post-exposure to a laboratory stressor. Regarding SBP recovery following stress exposure, six out of twelve studies reported quicker SBP recovery to baseline with exercise compared to control. Improvements were seen in studies employing ACE (n = 3), HIIE (n = 1), FE (n = 2) and RE + ACE (n = 1) protocols, with intensities ranging between light (n = 2), moderate (n = 2) and vigorous (n = 3). 

Similar to SBP, improvements in DBP recovery were seen in 6 out of 12 studies employing ACE (n = 4), FE (n = 2) and RE + ACE (n = 1) protocols with intensities ranging between light (n = 2), moderate (n = 2) and vigorous (n = 3). On the other hand, MAP recovery was assessed in only three studies, but no significant reductions were reported. Measurement of HR recovery was not commonly reported as HR reactivity, with only four of eight studies (intensity: moderate n = 3, vigorous n = 2; protocol: ACE n = 4, HIIE n = 1) showing a quicker return to baseline following stress exposure with exercise compared to control. Interestingly, Alderman et al. [11] found both moderate- and vigorous-intensity exercise improved HR recovery; however, vigorous-intensity exercise had a significantly greater effect than moderate intensity.

#### 3.5.6. Heart Rate Variability (HRV)

From our findings, the measurements of HRV were not as popular as other cardiovascular outcomes. There were only six studies (intensity: light n = 1, moderate n = 4, vigorous n = 3, near-maximal to maximal n = 1) that assessed stress-induced HRV responses, but none of them reported improvements in HRV following exercise when compared to control or pre-exercise. The methods of analyzing HRV in these studies were also inconsistent, whereby some studies analyzed frequency domain data while others used time domain data.

## 4. Discussion

### 4.1. Key Findings

In this scoping review, we specifically focused on the effects of acute or single exercise sessions of various modalities and intensities on stress-induced cardiovascular outcomes, namely BP and HR reactivity and recovery, as well as HRV in the general populations. BP reactivity was the most frequently studied outcome in these studies, followed by HR reactivity. From the total of 36 studies, we identified 17–20 articles which have reported lower BP reactivity with exercise compared to control. Overall, the majority of studies employed aerobic continuous exercise (ACE) with moderate intensity, while the high-intensity interval exercise (HIIE) modality is gaining interest in the field of exercise and stress-induced cardiovascular reactivity. 

Our findings showed that there seems to be a consistent trend pointing towards lower BP reactivity with acute exercise in response to a stress exposure, in particular with ACE modality of moderate intensity. This is consistent with a previous meta-analysis which examined the effects of acute aerobic exercise on BP reactivity to stress [19]. Other modalities such as HIIE and resistance exercise are also associated with reduced BP reactivity to stress to a lesser extent. Comparisons between exercise intensities or modalities are rather inconclusive; however, some of the findings seem to be suggesting that exercise of higher intensity [11,44,48] or volume [28,37] attenuates BP reactivity to a greater degree than exercise of low intensity or volume of the same modality. This may be related to a greater stimulation of endothelium-dependent vasodilation in humans through the increased production of nitric oxide, which may help to lower blood pressure responses during and post-exercise [51]. Interestingly, studies involving hypertension [9,25,51] or individuals with elevated BP [29,44,48] also showed favorable outcomes in BP reactivity with exercise, suggesting that those with an apparent cardiovascular risk factor may also benefit from the effects of acute exercise on reducing adverse BP responses from stress exposure. 

While there is evidence that acute exercise is effective at lowering BP reactivity, the findings around HR reactivity seem to suggest otherwise. Though a majority of studies examined HR reactivity to stress with exercise, very few studies demonstrated significantly positive outcomes. The lack of findings could be attributed to several factors, such as the exercise intensity and timing of stress exposure in relation to exercise completion. It is plausible that in some studies, HR responses from a prior exercise session had not returned to baseline levels before participants were exposed to a stressor, thus interfering with the actual HR response during exposure. In such cases, participants with lower cardiorespiratory fitness may need longer times for HR to recover to baseline after cessation of exercise. Furthermore, there is no denying that measuring HR during a stress exposure can be challenging, as body movements, optical noise and types of measuring device or wearables can impact the accuracy of HR measurements. The effects of acute exercise on BP and HR recovery remain inconsistent and may explain the variation in findings by the various timing of measurement in the recovery period following removal of a stressor, making it challenging to draw reliable conclusions.

### 4.2. Research Gaps and Implications for Future Research

Based on this review, we identified several relevant gaps that currently exist in the literature and can be avenues for further research. First, with regards to cardiovascular outcome measurements, our scoping review identified only six papers that investigated the effects of exercise on stress-induced HRV responses. HRV has recently gained attention in research on exercise and performance, and most of the studies cited in this review were conducted after 2017. HRV represents the cardiac autonomic function in response to a variety of internal and external stressors. A low HRV is associated with impaired autonomic control, which reduces the body’s ability to cope with stressors [52]. Certainly, this can be an outcome of interest to explore in future investigations related to exercise and autonomic reactivity. Second, comparisons between exercise modalities are rather inconclusive, as there was a huge variability in protocol durations and intensities across studies, which makes it challenging to draw reliable conclusions. However, there is one observation that is worth noting, and that is that the HIIE modality may be equally effective if not better than ACE in attenuating BP reactivity. The fact that there is growing and robust evidence that HIIE shows similar or greater efficacy compared with moderate-intensity continuous training across a range of cardiovascular outcomes [52,53,54,55] highlights the need for further research to dissect the benefits of this particular exercise in modulating BP responses to stress. Furthermore, HIIE can be performed in various ways by manipulating variables such as exercise modality, intensity, work interval, rest times and set repetitions, making it a flexible training program that can be designed to suit specific populations. In particular, it remains to be ascertained which HIIE protocol is the best in minimizing cardiovascular stress reactivity. Although the evidence in this review is limited, the findings somewhat suggest that shorter-bout protocols (<60 s) [13,29,41] may be associated with lower cardiovascular reactivity compared to longer-lasting bouts (>60 s) [21,30,46]. This hypothesis is corroborated by recent studies showing that longer HIIT protocols induced sympathetic hyperactivity and larger increases in cardiovascular responses compared to shorter bouts and moderate-intensity exercises [56,57]. This is certainly an area worthy of further investigation in helping to design safer HIIE/HIIT protocols that can minimize cardiovascular stress, especially for populations with chronic stress or elevated BP.

Third, a small number of studies in this review were conducted in hypertensive individuals and showed favorable outcomes on cardiovascular reactivity. While these findings are encouraging, more research is warranted to explore the benefits of acute exercise in mitigating stress-induced reactivity in this population as well as in those with the presence of multiple metabolic risk factors that can somewhat blunt a healthy cardiovascular response to stress. This has important implications for exercise as an effective therapy in combatting the deleterious impact of chronic stress and other risk factors that accelerate CVD. In addition, studies exploring how acute exercise can modulate molecular mediators such as muscle-derived myokines [58], and their possible cardioprotective role in mediating biological pathways involved in cardiovascular functions and stress reactivity, especially in populations with apparent cardiovascular risks, should also be considered.

Finally, could it be possible that the timing of exercise and stress exposure relative to the 24 h circadian cycle has an influence on reactivity of the cardiovascular system? It is established that BP and HR exhibit diurnal variations over a 24 h period, and these changes are under the influence of behavioral, humoral and autonomic (sympathetic nervous system) factors [59,60]. Given that it is evident that the sympathetic tone is greater in the morning compared to evening [61,62], perhaps investigating the influence of timing of exercise, e.g., morning vs. evening, on cardiovascular reactivity would shed some understanding regarding timing effect and cardiovascular health.

### 4.3. Study Limitations

While the findings of this review are encouraging, there are several limitations that should be acknowledged. First, given that this was a scoping review, we did not attempt to undertake quality appraisal of the included studies, therefore our findings cannot be geared at providing exercise recommendations that are best for lowering cardiovascular reactivity. Second, the selected studies involved populations with a wide range of age, body weight status and cardiorespiratory fitness. More importantly, there was a wide variation in the study protocols pertaining to exercise intensity, modality, types of stressors involved and the outcome measurements. Determining exercise intensity in some studies also proved to be a challenge due to the different indicators used (HR, power output, oxygen consumption, etc.). Thus, the heterogeneity of the studies precluded the generalization of our findings. Third, we categorized the exercise modalities based on four basic types of modalities for easier charting process, though we acknowledge that the classification strategy could very well extend beyond just four modalities. Finally, we also acknowledge that a large number of studies involved healthy, young adult participants, which may limit the generalization of these findings to older-aged groups and those with cardiovascular or metabolic diseases.

## 5. Conclusions

This review is the most recent to comprehensively report the breadth of literature on the effects of acute exercise on stress-induced cardiovascular reactivity. We conclude that exaggerated blood pressure responses to a stress stimulus can be attenuated with an acute bout of exercise prior to stress exposure. Although the majority of studies reported a positive outcome with aerobic continuous exercise of moderate intensity, a general consensus is hard to establish due to heterogeneity of exercise protocols and participant characteristics. Knowledge gaps concerning comparisons between exercise modalities or intensities, as well as timing of exercise and stress exposure in relation to the 24 h circadian cycle, may provide insight in understanding the therapeutic scope and effectiveness of acute exercise in managing stress responses to address the ill effects of stress on cardiovascular health.

## Figures and Tables

**Table 1:** Characteristics and outcomes of included studies.

Author	Study Design	Participants	Age (Years)	Exercise			Stressor		Outcome Parameters	Main Findings
				Type	Intensity	Duration	Type	Time of Measurement		
Aladro-Gonzalvo et al., 2019 [21]	Crossover RCT	25M Physically active and inactive	R: 12–15	HIIE (interval cycling)	Vigorous	26 min	Social (TSST-C)	5 min post-exercise	SBP, DBP, HR, HRV	Better SBP and HR recovery with exercise. No changes to BP, HR and HRV reactivity.
Alderman et al., 2007 [11]	Parallel RCT	48M/42F Healthy, non-smoker, physically active	M: 22.9 ± 4.2 R: 18–34	ACE (treadmill)	(1) Moderate (2) Vigorous	30 min	Cognitive (MA)	5, 30 or 60 min post-exercise	SBP, DBP, HR	Lower SBP, DBP and HR reactivity, and better HR recovery after both exercises. DBP recovery improved only after vigorous-intensity ACE. Vigorous-intensity ACE has greater effect on SBP reactivity, HR reactivity and HR recovery.
Bartholomew 2000 [22]	Parallel RCT	17M/23F Competitive athletes	M: 21.2 R: 18–25	ACE (treadmill)	Near-maximal to maximal	Not applicable	Cognitive (MA) Social (Stroop, Speech)	40 min post-exercise	MAP, HR	Lower MAP reactivity with exercise. No changes to HR reactivity.
Benvenutti et al., 2017 [23]	Crossover RCT	13M/11F Healthy, non-smoker	M: 22.9 ± 3.5	FE (yoga)	-	30 min	Cognitive (MA)	Immediately post-exercise	SBP, DBP, HR, HRV	Better SBP and DBP recovery with exercise. No changes to SBP, DBP, HR and HRV reactivity.
Boone et al., 1993 [9]	Crossover RCT	8 (gender unknown) Hypertension, otherwise healthy	M: 41.1 ± 6.5	ACE (treadmill)	Moderate	60 min	Cognitive (Stroop)	10 min post-exercise	SBP, DBP, MAP, HR	Lower SBP, DBP and MAP reactivity with exercise. No changes to HR reactivity.
Brownley et al., 2003 [10]	Pre–post trial	12M/12F Healthy, sedentary	M: 24.5 ± 4.0	ACE (cycling)	Moderate	25 min	Cognitive (MA) Social (Speech)	Pre- and 30 min post-exercise	SBP, DBP, MAP, HR	Lower SBP, DBP and MAP reactivity with exercise. No changes to HR reactivity.
Ebbesen et al., 1992 [24]	Parallel RCT	24M Healthy, sedentary	R: 18–35	ACE (cycling)	Moderate	(1) 1 h (2) 2 h	Physiological (CPT), Cognitive (Stroop), and Social (Speech)	1, 3 and 24 h post-exercise	SBP, DBP, HR	Lower SBP reactivity with 1 h ACE. Lower DBP reactivity with both exercises. No changes to HR reactivity and recovery.
Farah et al., 2021 [13]	Crossover RCT	13M Healthy, non-smoker, physically inactive	M: 22.8 ± 2 R: 21–28	(1) HIIE (body weight) (2) ACE (treadmill)	(1) Vigorous (2) Moderate	(1) 20 min (2) 30 min	Physiological (CPT)	30 min post-exercise	SBP, DBP, MAP, HR	Lower SBP, DBP, MAP reactivity with HIIE. Lower SBP reactivity reduced with ACE. No changes to BP recovery, HR reactivity and HR recovery. No differences between HIIE and ACE on SBP reactivity.
Gauche et al., 2017 [25]	Pre–post trial	10F Hypertension otherwise healthy	M: 71.1 ± 5.5	(1) RE(t) (2) RE(c)	Moderate	Not applicable	Cognitive (Stroop)	Pre- and 60 min post-exercise	SBP, DBP, MAP, HRV	Lower SBP, DBP, MAP and HRV (In LF) reactivity with exercise. No differences between RE(t) and RE(c) on SBP, DBP and MAP reactivity.
Hamer et al., 2006 [26]	Crossover RCT	30M Healthy, non-smoker	M: FH+: 22 ± 0.4FH−: 21 ± 0.6	ACE (cycling)	Vigorous	20 min	Cognitive (Stroop)	30 min post-exercise	SBP, DBP, HR	Lower HR reactivity with exercise. No changes to SBP and DBP reactivity.
Heffernan et al., 2017 [27]	Crossover RCT	9M/6F Healthy, non-smoker	M: 26 ± 1	RE	Light-to-moderate	30 min	Physiological (CPT)	30 min post-exercise	SBP, DBP	Lower DBP reactivity with exercise. No changes to SBP reactivity, SBP recovery and DBP recovery.
Hobson and Rejeski 1993 [28]	Parallel RCT	80F 3 smokers	M: 18.3 ± 0.9	ACE (cycling)	Vigorous	(1) 10 min (2) 25 min (3) 40 min	Cognitive (Stroop)	20 min post-exercise	SBP, DBP, MAP, HR	Lower DBP and MAP reactivity with 40 min ACE.
Ketelhut et al., 2016 [29]	Pre–post trial	39M Healthy, non-smoker	M: 34 ± 8	HIIE (cycling)	Near-maximal to maximal	30 min	Physiological (CPT)	Pre- and 60 min post-exercise	SBP, DBP	Lower SBP and DBP reactivity with exercise.
Meireles et al., 2020 [30]	Crossover RCT	22M Healthy, non-smoker, physically inactive	M: 23 ± 2	(1) HIIE (cycling) (2) ACE (cycling)	(1) Vigorous (2) Moderate	(1) 20 min (2) 30 min	Physiological (CPT)	30 min post-exercise	SBP, DBP, HR, HRV	Lower HR reactivity after ACE. No changes to BP and HRV reactivity. No differences between HIIE and ACE on HR reactivity.
Messerli-Bürgy et al., 2019 [31]	Parallel RCT	NW-con: 6M/7F NW-ex: 7M/6F OW/OB-con: 6M/6F OW/OB-ex: 3M/9F Healthy	M: NW-con: 8.5 ± 0.9 NW-ex: 8.6 ± 0.6 OW/OB-con: 9.6 ± 1.4 OW/OB-ex: 8.9 ± 1.2	ACE (basketball, running)	Moderate	30 min	Social (TSST-C)	15 min post-exercise	SBP, DBP, HR	Better HR recovery with exercise. No changes to BP reactivity and recovery.
Monroe et al., 2018 [32]	Crossover RCT (pilot study)	9F Healthy, physically active	R: 20–33	(1) FE (yoga) (2) ACE (cycling)	(1) Very light to light (2) Light-to-moderate	20 min	Physiological (CPT)	20 min post-exercise	SBP, DBP, HR	Better SBP and DBP recovery with both exercises. No changes to BP and HR reactivity. No differences between FE and ACE on BP recovery.
Moreira et al., 2014 [33]	Crossover RCT	10M/10F Healthy	M: 33.4 ± 6.9 R: 24–50	RE + ACE	Moderate-to-vigorous	Not applicable	Physiological (CPT)	Pre- and 60 min post-exercise	SBP, DBP	Lower BP reactivity and better recovery with exercise.
Neves et al., 2012 [34]	Crossover RCT	Con (subgroup): 2M/9F Ex: 4M/22F Healthy, non-smoker, sedentary	M: Con: 28 ± 2 Ex: 29 ± 8	ACE (treadmill)	Near-maximal to maximal	≈10 min	Cognitive (Stroop)	Pre- and 1 h post-exercise	SBP, DBP, MAP, HR	Lower SBP reactivity with exercise. No changes to DBP, MAP and HR reactivity.
Paine et al., 2013 [35]	Crossover RCT	18M Healthy	M: 20.4 ± 1.2	RE (leg extension)	Vigorous	Not applicable	Cognitive (MA)	6 h post-exercise	SBP, DBP, HR, RMSSD	No changes to BP, HR and HRV reactivity.
Probst et al., 1997 [36]	Crossover RCT	12M/12F Not highly trained, average physical fitness	M: 23.3 ± 0.7 R: 19–26	ACE (cycling)	Moderate	30 min	Cognitive (Stroop), Physiological (CPT)	Pre- and 15 min post-exercise	SBP, DBP, MAP, HR	Lower SBP and HR reactivity with exercise. No changes to DBP and MAP reactivity.
Rejeski et al., 1991 [37]	Crossover RCT	12 (gender unknown) Trained cyclists, physically active	M: 30.6 ± 1.5 R: 23–38	ACE (cycling)	(1) Moderate (2) Vigorous	(1) 30 min (2) 60 min	Cognitive (Stroop)	30 min post-exercise	SBP, DBP, MAP, HR	Lower DBP and MAP reactivity with both exercises. No changes to HR reactivity. Only vigorous-intensity ACE reduced SBP reactivity. Vigorous-intensity ACE has greater effect on SBP and MAP reactivity.
Rejeski et al., 1992 [38]	Crossover RCT	48F	R: 25–40	ACE (cycling)	Vigorous	40 min	Cognitive (Stroop) Social (Speech)	30 min post-exercise	SBP, DBP, MAP, HR	Lower SBP, DBP and MAP reactivity with exercise. No changes to HR reactivity.
Rejeski et al., 1995 [39]	Parallel RCT	8M/20F Knee osteoarthritis, on medications, sedentary	M: ACE: M: 72 ± 8.1 F: 68.6 ± 6.0 RE: M: 65.0 ± 4.3 F: 69.6 ± 4.8	(1) ACE (walking) (2) RE	(1) Moderate-to-vigorous (2) -	40 min	Cognitive (MA)	15 min post-exercise	SBP, DBP, HR	No changes to SBP, DBP and HR reactivity.
Rocha et al., 2012 [40]	Pre–post trial	GG: 3M/13F GT/TT: 4M/15F 894G > T polymorphism otherwise healthy, non-smoker, sedentary	R: 18–49 M: GG: 34 ± 15 GT/TT: 28 ± 20	ACE (treadmill)	Near-maximal to maximal	≈10 min	Cognitive (Stroop)	Pre- and 60 min post-exercise	SBP, DBP, HR	Lower DBP and HR reactivity in GT/TT group. Only DBP reactivity lower in GG. No changes to SBP reactivity.
Roemmich et al., 2009 [41]	exp1: Parallel RCT	Con: 7/7 Ex: 7/7 Healthy	M: Con: 10.8 ± 0.8 Ex: 10.4 ± 1.3	HIIE (cycling)	Vigorous	20 min	Social (Speech)	20 min post-exercise	SBP, DBP, HR	Lower DBP reactivity with exercise. No changes to SBP and HR reactivity.
Roemmich et al., 2009 [41]	exp2: Crossover RCT	11M/11F Healthy	M: 10.5 ± 1.4	HIIE (cycling)	Vigorous	20 min	Social (Speech)	20 min post-exercise	SBP, DBP, HR	Lower SBP, DBP and HR reactivity with exercise.
Rooks et al., 2011 [42]	Crossover RCT	24F Healthy, 11 smokers, inactive	Smoker: M: 20.6 ± 2.1 R: 18–26 Non-smoker: M: 20.5 ± 2.3 R: 18–26	ACE (cycling)	Moderate	30 min	Physiological (CPT), Cognitive (Stroop)	Pre- and 10 min post-exercise	HR	No changes to HR reactivity.
Roth 1989 [43]	Parallel RCT	40M/40F Healthy, physically active and inactive	M: 20.8 ± 3.5	ACE (cycling)	Light-to-moderate	20 min	Cognitive (Digits backward, MA)	Pre- and post-exercise	SBP, DBP, HR	No changes to SBP, DBP and HR reactivity. Females had greater HR reactivity than males.
Roy and Steptoe 1991 [44]	Parallel RCT	Con: 10M Light-intensity ACE: 10M Moderate-intensity ACE: 10M Healthy	M: Con: 21.1 ± 2.2 Light-intensity ACE: 20.7 ± 2.8 Moderate-intensity ACE: 21.3 ± 2.2	ACE (cycling)	(1) Light (2) Moderate	20 min	Cognitive (MA)	20 min post-exercise	SBP, DBP, HR	Lower SBP, DBP and HR reactivity, better SBP, DBP and HR recovery with moderate-intensity ACE. Moderate-intensity ACE had greater effect on SBP recovery, HR reactivity and HR recovery.
Santaella et al., 2006 [45]	Crossover RCT	NT: 8M/6F HT: 10M/6F Healthy, sedentary	M: 39 ± 2	ACE (cycling)	Moderate	45 min	Cognitive (Stroop)	80 min post-exercise	SBP, DBP	Lower DBP reactivity with exercise. No changes to SBP reactivity.
Scott et al., 2008 [46]	Crossover RCT	10M Competitive cyclist or triathletes, non-smokers, physically active	M: 29 ± 2 R: 21–37	(1) HIIE (cycling) (2) ACE (cycling)	Near-maximal to maximal and moderate	(1) 60 min (2) 50.1 ± 5.2 min	Physiological (LBNP)	20 min post-exercise	MAP, HR, HRV	No changes to MAP, HR and HRV reactivity and recovery. No differences between HIIE and ACE.
Someya et al., 2012 [47]	Crossover RCT	11M Healthy	M: 25 ± 5	ACE (cycling)	Moderate	30 min	Cognitive (MA)	15 min post-exercise	SBP, DBP, MAP	No changes to SBP, DBP and MAP reactivity and recovery.
Steptoe et al., 1993 [48]	Parallel RCT	72M Competitive amateur athletes and inactive men	R: 20–35 M: Sportsmen: 24.4 ± 3.6 Inactive men: 27.3 ± 4.1	ACE (cycling)	(1) Moderate (2) Vigorous	20 min	Cognitive (MA), Social (Speech)	30 min post-exercise	SBP, DBP, HR	Lower SBP reactivity and better SBP and DBP recovery with vigorous intensity. No changes to HR reactivity.
Taylor and Katomeri 2006 [49]	Parallel RCT	Con: 15M/14F Ex: 11M/20F Healthy, smoker	M: Con: 30.1 ± 9.7 Ex: 27.1 ± 5.5	ACE (brisk walk)	Very light	15 min	Cognitive (Stroop) Social (Speech)	10 min post-exercise	SBP, DBP, MAP, HR	Lower SBP, DBP and MAP reactivity with exercise. No changes to HR reactivity.
Taylor and Oliver 2009 [12]	Crossover RCT	5M/25F physically active	M: 25.3 ± 9.7	ACE (brisk walk)	Light	15 min	Cognitive (Stroop,)	10 min post-exercise	SBP, DBP, MAP	Lower SBP, DBP and MAP reactivity with exercise.
Vianna et al., 2014 [50]	Pre–post trial	15M/19F Healthy	M: M: 25 ± 2 F: 24 ± 1	ACE (treadmill)	Near-maximal to maximal	Not applicable	Cognitive (Stroop)	Pre- and 60 min post-exercise	MAP, HR	Lower MAP reactivity. No changes to HR reactivity.
West et al., 1998 [51]	Crossover RCT	14M/18F 21 normotensive, 11 hypertensive sedentary	M: 34 ± 8 R: 21–47	ACE (cycling)	Moderate	20 min	Physiological (CPT), Cognitive (MA)	20 min post-exercise	SBP, DBP, MAP	Lower DBP reactivity with exercise. No changes to SBP and MAP reactivity.

ACE, aerobic continuous exercise; Con, control group; CPT, cold pressor task; DBP, diastolic blood pressure; Ex, exercise group; F, female; FH+, with family history of hypertension; FH−, without family history of hypertension; FE, flexibility exercise; GG, without 894G > T polymorphism in nitric oxide synthase; GT/TT, with 894G > T polymorphism in nitric oxide synthase; HIIE, high-intensity interval exercise; h, hour; HR, heart rate; HRV, heart rate variability; HT, hypertensive; LBNP, lower-body negative pressure; In LF, logarithmic normalization of low frequency; M (for age), mean; M (for gender), male; MA, Mental arithmetic task; MAP: mean arterial pressure; mins, minutes; NT, normotensive; NW, normal weight; OB, obese; OW, overweight; R, range; RCT, randomized control trial; RE, resistance exercise; RE(t), traditional resistance exercise; RE(c), circuit-based resistance exercise; RMSSD, root mean square of successive differences; SBP, systolic blood pressure; TSST-C, Trier Social Stress Test for children.

## Data Availability

Not applicable.

## Appendix A

**Table A1:** PRISMA-ScR Checklist.

Section	Item	Prisma-Scr Checklist Item	Reported on Page
**Title**			
Title	1	Identify the report as a scoping review.	1
**Abstract**			
Structured summary	2	Provide a structured summary that includes (as applicable): background, objectives, eligibility criteria, sources of evidence, charting methods, results and conclusions that relate to the review questions and objectives.	1
**Introduction**			
Rationale	3	Describe the rationale for the review in the context of what is already known. Explain why the review questions/objectives lend themselves to a scoping review approach.	1–2
Objectives	4	Provide an explicit statement of the questions and objectives being addressed with reference to their key elements (e.g., population or participants, concepts and context) or other relevant key elements used to conceptualize the review questions and/or objectives.	2
**Methods**			
Protocol and registration	5	Indicate whether a review protocol exists; state if and where it can be accessed (e.g., a Web address); and if available, provide registration information, including the registration number.	-
Eligibility criteria	6	Specify characteristics of the sources of evidence used as eligibility criteria (e.g., years considered, language and publication status), and provide a rationale.	3
Information sources	7	Describe all information sources in the search (e.g., databases with dates of coverage and contact with authors to identify additional sources), as well as the date the most recent search was executed.	3
Search	8	Present the full electronic search strategy for at least 1 database, including any limits used, such that it could be repeated.	3
Selection of sources of evidence	9	State the process for selecting sources of evidence (i.e., screening and eligibility) included in the scoping review.	3
Data charting process	10	Describe the methods of charting data from the included sources of evidence (e.g., calibrated forms or forms that have been tested by the team before their use, and whether data charting was conducted independently or in duplicate) and any processes for obtaining and confirming data from investigators.	3
Data items	11	List and define all variables for which data were sought and any assumptions and simplifications made.	2
Critical appraisal of individual sources of evidence	12	If carried out, provide a rationale for conducting a critical appraisal of included sources of evidence; describe the methods used and how this information was used in any data synthesis (if appropriate).	-
Synthesis of results	13	Describe the methods of handling and summarizing the data that were charted.	3
**Results**			
Selection of sources of evidence	14	Give numbers of sources of evidence screened, assessed for eligibility and included in the review, with reasons for exclusions at each stage, ideally using a flow diagram.	4
Characteristics of sources of evidence	15	For each source of evidence, present characteristics for which data were charted and provide the citations.	5–15
Critical appraisal within sources of evidence	16	If carried out, present data on critical appraisal of included sources of evidence (see item 12).	-
Results of individual sources of evidence	17	For each included source of evidence, present the relevant data that were charted that relate to the review questions and objectives.	16
Synthesis of results	18	Summarize and/or present the charting results as they relate to the review questions and objectives.	16–18
**Discussion**			
Summary of evidence	19	Summarize the main results (including an overview of concepts, themes and types of evidence available), link to the review questions and objectives and consider the relevance to key groups.	18–19
Limitations	20	Discuss the limitations of the scoping review process.	20
Conclusions	21	Provide a general interpretation of the results with respect to the review questions and objectives, as well as potential implications and/or next steps.	20
**Funding**			
Funding	22	Describe sources of funding for the included sources of evidence, as well as sources of funding for the scoping review. Describe the role of the funders of the scoping review.	20
